# Social Attitudes toward Cerebral Palsy and Potential Uses in Medical Education Based on the Analysis of Motion Pictures

**DOI:** 10.1155/2015/341023

**Published:** 2015-07-14

**Authors:** Marek Jóźwiak, Brian Po-Jung Chen, Bartosz Musielak, Jacek Fabiszak, Andrzej Grzegorzewski

**Affiliations:** ^1^Department of Pediatric Orthopedics and Traumatology, Poznań University of Medical Sciences, 61-545 Poznań, Poland; ^2^Faculty of English, Adam Mickiewicz University in Poznań, 61-874 Poznań, Poland; ^3^Department of Orthopedics and Pediatric Orthopedics, Medical University of Łódź, 91-002 Łódź, Poland

## Abstract

This study presents how motion pictures illustrate a person with cerebral palsy (CP), the social impact from the media, and the possibility of cerebral palsy education by using motion pictures. 937 motion pictures were reviewed in this study. With the criteria of nondocumentary movies, possibility of disability classification, and availability, the total number of motion pictures about CP was reduced to 34. The geographical distribution of movie number ever produced is as follows: North America 12, Europe 11, India 2, East Asia 6, and Australia 3. The CP incidences of different motor types in real world and in movies, respectively, are 78–86%, 65% (Spastic); 1.5–6%, 9% (Dyskinetic); 6.5–9%, 26% (Mixed); 3%, 0% (Ataxic); 3-4%, 0% (Hypotonic). The CP incidences of different Gross Motor Function Classification System (GMFCS) levels in real world and in movies, respectively, are 40–51%, 47% (Level I + II); 14–19%, 12% (Level III); 34–41%, 41% (Level IV + V). Comparisons of incidence between the real world and the movies are surprisingly matching. Motion pictures honestly reflect the general public's point of view to CP patients in our real world. With precise selection and medical professional explanations, motion pictures can play the suitable role making CP understood more clearly.

## 1. Introduction

The media have a tremendous impact on the creation of images of people. Depictions of disability in motion pictures play a major role in forming public perceptions of disability.

Artists have faced the problem of portraying disabilities for ages. Works of art representing movement disorders were produced in ancient times. With the rise of new eras of art, new ideas were developed for depicting physical disabilities. Greek mythology presented physical disabilities as a punishment from the gods. The New Testament portrayed disability as suffering that purifies the soul and shapes the human personality. In the Renaissance, disabilities such as Saint Vitus' dance (choreomania) were portrayed in art. The onset of motion pictures produced new ways of illustrating movement disorders. Cinematography dates to the end of the 19th century and the beginning of the 20th century, and the first movie focusing on disabilities was released during this time: a film with rather crude humor titled “The Fake Beggar,” produced in 1898 by the famous American inventor Thomas Alva Edison [1]. The short film (with a runtime of less than 1 minute) presents a “legless” man begging on the street who stands to pick up a coin that misses his cup and is discovered and chased by a policeman. In the early days of cinematography, interest in the problems of disabled people was generally marginal. Only a few movies from the first half of the 20th century addressed this issue, and these films attracted controversy after their release. For example, “Freaks,” by Tod Browning in 1932, was banned in some countries due to the negative perception of disabled people in these societies [2]. The attitudes of the public and the audiovisual media toward people with disabilities have evolved, and anxiety, marginalization, and rejection have been replaced with compassion and acknowledgment (which may also be negative). Attitudes are slowly changing to acceptance and understanding. However, the media continue to portray people with disabilities in a stigmatizing way using stereotypical approaches, such as pity and heroism. Since the end of the 1980s (after the release of the excellent film “My Left Foot,” the touching story of Irish writer Christy Brown), there has been an increase in the production of movies that address physical and intellectual disabilities. Moreover, these have not been minor productions; among them can be found major titles, such as “Music Within” (2007), “Door to Door” (2009), and “What's Eating Gilbert Grape” (1993). The number of films released thus far is significant. This study presents a systematic review of movies about cerebral palsy (CP) and movement disorders.

## 2. Materials and Methods

The movies in this study were obtained mainly from The Internet Movie Database (IMDb) [3] and FilmWeb [4]. Additional information was obtained from articles addressing the issue of disability in motion pictures [1, 2, 5]. IMDb is an online database of information related to movies, television shows, actors, production crew personnel, video games, and fictional characters featured in visual entertainment media. It is the largest existing film database containing movie plots and trailers (with more than 1.5 million movie titles as of 2009). Keywords used to scan the databases were directly and indirectly related to definitions of CP, physical disability, and movement disorders. The following keywords were used to search the movie databases: disability, physical disability, handicapped, paralysis, palsy, cerebral palsy, spasticity, dyskinesia, ataxia, athetosis, muscular dystrophy, and chorea. The total number of movies evaluated in this study was 937. In the first stage of the study, the review of motion pictures was based on the evaluation of movie plots and movie trailers. The subsequent analysis examined parts of movies and full-length movie files. Movies that did not meet the following selection criteria were excluded: movies classified as fictional or TV series; characters in the movies that presented any type of movement disorder that was sufficiently extensive or detailed to determine the type of disorder; and movie files that were available to watch.

**Figure 1:**
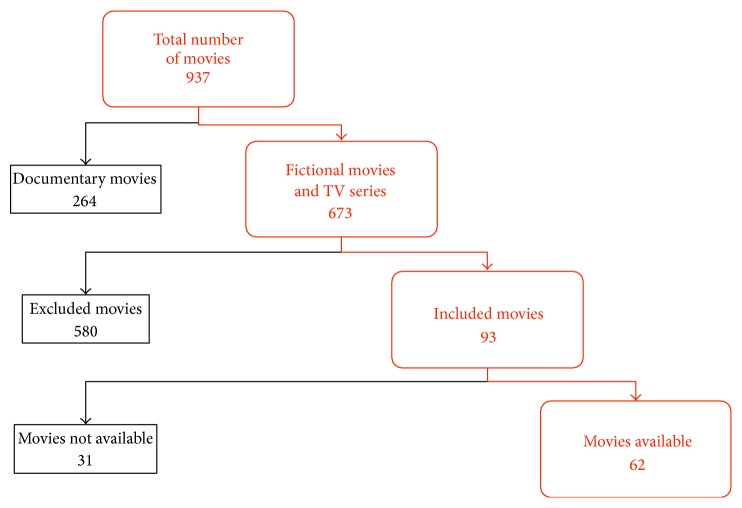
Movie selection process. Criteria with the number of movies included (on the right, in red) and excluded (on the left, in black) at each stage.

Movies that focused on the problems of people with intellectual disabilities or that were classified as documentaries were also excluded. These criteria resulted in a final analysis of 62 movies (Figure 1). All movie files were analyzed in terms of the quality of the presentation of medical signs of disorders and the exposition of the social and psychological problems of disabled people. Additional information was recorded, such as the year and country of production and the movie genre. Movies were classified by the type of disorder. Movies with essential plot elements concerning people with CP constituted the major part of the research. Characters from this group were categorized based on the type of motor disability (spastic, dyskinetic, mixed, ataxic, or hypotonic) and the Gross Motor Function Classification System (GMFCS) level. The incidence of particular motor disabilities and GMFCS levels were counted among the movie characters with CP, and the results were compared with the incidence in the real world [6, 7]. The collected films were also distinguished by the country of origin and time period.

## 3. Results

Our selection was narrowed from 62 movies about all types of movement disorders to 34 movies that specifically focused on roles with CP (Table 1). We categorized these movies using several different criteria: timeline, geographical distribution, type of motor disability, and the GMFCS level.

### 3.1. Timeline of CP Movies

We categorized the movies into five-year intervals from 1980 to 2011. The results are shown in Table 2.

### 3.2. Geographical Distribution

**Figure 2:**
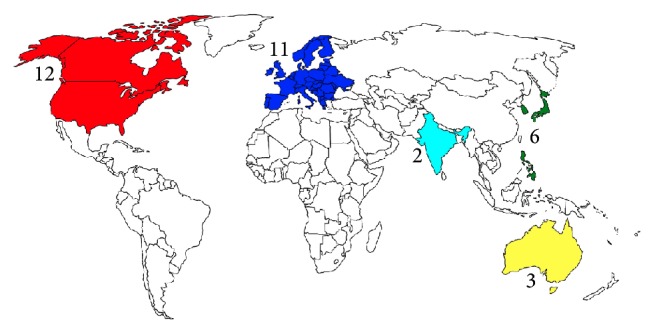
The geographic distribution of CP movies. Each color represents a specific geographical area or country: North America (red), Europe (blue), India (cyan), North-East and South-East Asia (green), and Australia (yellow).

To understand the differences between the geographic areas, we categorized the 34 movies about CP by their countries of production. The distribution is shown on the world map (Figure 2).

### 3.3. Incidence of CP by Motor Types

According to two major studies by Howard et al. [6] and Gorter et al. [7], CP has been divided into the following motor types: spastic, dyskinetic, mixed, ataxic, and hypotonic. The real world incidence of each motor type was identified in both previous studies (Table 3). We categorized the selected 34 CP movies by these motor types. The results are shown in Table 4.

### 3.4. Incidence of CP by GMFCS Levels

GMFCS is a five-level classification system developed by CanChild Centre for Childhood Disability Research at McMaster University in Canada [8]. The classification system describes the gross motor function of children and youth with CP on the basis of their self-initiated movement, with particular emphasis on sitting, walking, and wheeled mobility. The original version was developed in 1997. As of 2007, the expanded and revised version (GMFCS-E&R) includes an age band for youth 12 to 18 years old.

The real world incidence of GMFCS levels has been mentioned in studies by Howard et al. [6] and Gorter et al. [7] (Table 5). We categorized our selection of movies by GMFCS levels. The results are shown in Table 6.

## 4. Discussion

The aim of this paper was to present the social attitude toward the disabled persons with CP. Patients with this disease constitute a specific group of movement disorders; they suffer from the disease through their whole life; the character of their motor impairment is not progressive and they represent the wide spectrum of movement and intellectual dysfunctions. This makes the disease one of the most representatives for physical disability. Among other movement disorders which are commonly found in media, only paralysis with different origins is more frequent then CP. However, functional impairment and hence the community attitude to medical and social problems of these patients are different from what we see in patients with CP. Thus, we decided to focus only on the group of individuals who suffer from CP.

### 4.1. Timeline of CP Movies

Before 1990, the movie industry showed little interest in the subject of CP. Only one movie, “I, Claudius” (1976), included a character with CP before 1980 (Table 2). Worldwide interest in this topic has increased since 1990. The subject of CP has been a significant focus, as reflected by the number of movies released during each time interval. These movies include major productions, such as “What's Eating Gilbert Grape” (1993), “The Usual Suspects” (1995), and “Music Within” (2007). We believe that both the development of the movie industry and the great success of the movie “My Left Foot” (1989) have significantly influenced the number of movies produced about CP, leading to the golden years of CP movies between 2000 and 2009. However, the number of these movies has decreased since 2005. Because of the significant progress in visual effect technologies during the 1990s, most movie productions have focused on more commercial and entertaining topics. Roles involving disabilities such as paralysis and amputation are used to demonstrate the possibilities of postproduction visual effects, but CP has again been moved to the sidelines.

### 4.2. Geographical Distribution

As shown in Figure 2, movies about CP are mainly produced in North America and Europe, with other productions in Eastern Asia, India, and Australia. The rest of the world either has no movie industry or does not focus on CP-related topics. In North America and Europe, movies about CP were primarily produced in the 1990s. Interestingly, during the past 5 years, most movies about CP were produced in Mid- and East Asia, including India, South Korea, and Japan. Asian societies remain relatively conservative. Among the general public of nonmedical professionals worldwide, CP has been treated as a troublesome disease or even an infectious disease for many years, and society has socially isolated these patients. Only very recently have the disease itself and the human rights of CP patients gradually begun to be understood. This may explain the shift in CP-related movie productions from Western to Eastern countries during the past several years. However, when the total number of movies is compared, the Asian movie industry does not focus significantly on this topic.

Based on the timeline and geographical distribution analysis, significant questions have arisen: What is the social attitude towards people with CP? Has it changed during last decades and does the public understand the disorder? The social attitude has significantly evolved during last several decades [9–11]. Physically disabled people evolved from bedridden to independent people. Common appearances of this population in public life made the society be more aware of their needs and change the attitude from curiosity and compassion to acceptance. This made the relationships between disabled and abled-body populations more balanced. Motion pictures presented here depict this evolution. Movies had significant contributions to raise the awareness of needs of disabled people.

We confirmed while discussing the needs and life comfort of CP individuals before and after watching selected movies from our list, such as “My Left Foot” (1989) and “Music Within” (2007), with different work groups representatives, including medical professionals and medicine students and teachers. The list of needs described before and after watching these movies was only partially consistent; for example, none of them listed sexual needs before watching, while all of them highlighted this problem as of high importance. Moreover, their general perceptions were changed from compassion and even fear to balanced acceptation and willingness to help.

### 4.3. Incidence of CP by Motor Types

The comparison of the incidence of CP in the real world (Table 3) and in movies (Table 4) suggested some correlations (Table 7). There are no movies about the ataxic and hypotonic types of CP. In contrast, movie roles with mixed types are more prominent than their real world incidence. A possible explanation for this finding is that screenplay writers and directors focus more on the psychological and social problems of CP than on the medical condition. Typical or stereotyped symptoms of the disease are often mixed rather than presented with medical precision. Because spasticity is the most dominant impression of CP patients among the general public, actors in CP roles often present this motor dysfunction.

### 4.4. Incidence of CP by GMFCS Levels

We compared the CP incidence of each GMFCS level between the real world (Table 5) and movies (Table 6). We found no correlations between these categories (Table 8).

**Figure 3:**
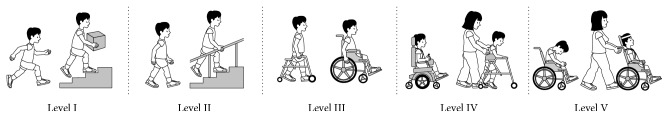
The Gross Motor Function Classification System (GMFCS). Permission to use the illustration was acquired from the copyright holder: Kerr Graham, Bill Reid, and Adrienne Harvey, The Royal Children's Hospital, Melbourne, Australia.

Medical professionals who are trained to use the GMFCS classification may classify a CP patient into a specific GMFCS level from I to V (Figure 3) [8]. To the general public, however, GMFCS I and II patients seem very similar to each other. Both groups have independent walking and transferring abilities. The GMFCS III category includes patients with any kind of handheld walking-assistance devices. Finally, patients with GMFCS levels IV and V are mostly confined to wheelchairs or beds. It was confirmed by asking three individuals from different medical specialties to assess the GMFCS level of characters in selected movies (Figure 3): With the five-level scale, the agreement was less than 50%. However, under the three-level scale, the agreement was almost 100%. Therefore, CP patients are generally grouped into three categories rather than five, as in the GMFCS classification. Based on the explanation above, we combined the GMFCS I and II categories and the GMFSC IV and V categories. The results are shown in Table 9. In this comparison, we can clearly see that the incidence in the real world and in movies is surprisingly similar.

### 4.5. Educational Purposes

In movies about CP, many great actors provide excellent representations of the typical symptoms of CP patients. We believe these movies are suitable materials for educational purposes. Interestingly, as we noticed, making the connection between students' personal experiences, such as motion pictures which they have watched or disease they are currently studying, can always facilitate their interests and help them to dig more into this specific disease. Also, pointing out the performance mistakes in the motion pictures helps students to realize more about true clinical symptoms which are related with the decision making process of the treatment. Below, we present some of the selected performances which could be useful for educational purposes.

An excellent presentation of the features of spasticity can be found in the South Korean movie “Oasis” (2002), directed by Chang-dong Lee. This is the story of a young former prisoner who maintains a friendship with a woman with CP despite a lack of acceptance from their families. Spasticity causes individuals to have decreased motor control. The main character in this film, Gong-ju, suffers from CP and has difficulty closing and opening her hands, which significantly reduces her manual abilities. She has difficulties with simple activities, such as using lipstick. In a few movie scenes, the typical alignment of a spastic hand can be seen. However, this is not a typical presentation of the spastic type of CP; some dystonic and athetotic movements overlap with general spasticity in these images [12]. Chorea can be explained using scenes from the movie “Music Within” (2007). One of the main characters in the movie, Art (played by Michael Sheen), is a person with fairly severe choreatic movements involving various parts of his entire body. The axial muscles, especially the muscles of the head and neck, are also involved, producing the characteristic grimace, which is presented often by this actor [13, 14]. Because of the involuntary movements that overlap with spasticity, this character cannot perform simple activities, such as opening a can or inserting a coin in a drink dispenser. “Music Within” is a worthwhile movie about Richard Pimentel, an actual disabled rights activist. This movie presents the problems of disabled people, societal attitudes towards them, and their limitations in daily activities. The movie “What's Eating Gilbert Grape” (1993) can be used to present athetosis. Arnie Grape, played by Leonardo DiCaprio, is the younger brother of the main character in the movie. He suffers from an intellectual disability and probably also from a mild form of CP. He presents writhing, sinuous, and slow movements, especially marked in the left extremity, mostly in the digits. His movements are similar to the definition of athetosis but seem to be exaggerated, leading to hyperextension in the joints [15]. It is worth noting that DiCaprio was nominated for an Oscar in the category of best supporting actor for this role. Some outstanding performances that offer excellent examples of the various GMFCS levels of CP are listed in Table 10.

## 5. Conclusions

Since the beginning of human history, the visual arts have mirrored the real world, in terms of both interpersonal relations and various social problems. CP has become an important issue. Hence, this study examines how this disease is reflected in a contemporary form of art, the motion picture. This type of media allows the general public to understand and acknowledge patients with CP.

## Figures and Tables

**Table 1:** List of motion pictures analyzed in this study.

Title	Year of production	Country of production	Type of movement disorder
39 Pounds of Love	2005	USA	Not CP
A Step Toward Tomorrow	1996	USA	Not CP
A Time to Live	1985	USA	Not CP
Aaltra	2004	Belgium	Not CP
Above Suspicion	1995	USA	Not CP
Angel	2011	India	Cerebral palsy
Annie's Coming Out	1984	Australia	Cerebral palsy
Bicycle Bobby	2009	USA	Cerebral palsy
Bitter Moon	1992	France	Not CP
Cousin	1999	Australia	Cerebral palsy
Dance Me To My Song	1998	Australia	Cerebral palsy
Door to Door [Japan]	2009	Japan	Cerebral palsy
Door to Door [USA]	2002	USA	Cerebral palsy
Everything That Rises	1998	USA	Not CP
Exo-Man	1977	USA	Not CP
Extras	2005	UK	Cerebral palsy
Flickan i Regnet	1955	Sweden	Not CP
Freaks	1932	USA	Cerebral palsy
Gaby: A True Story	1997	Mexico	Cerebral palsy
Good Luck	1996	USA	Not CP
Happy Go Lucky	2003	Hong Kong	Cerebral palsy
He Was A Quiet Man	2007	USA	Not CP
How To Kill Your Neighbor's Dog	2003	USA	Cerebral palsy
I, Claudius	1976	UK	Cerebral palsy
Inside I'm Dancing	2004	Ireland	Cerebral palsy
Interrupted Melody	1955	USA	Not CP
Juliets	2010	Taiwan	Not CP
La Chambre Des Magiciennes (Of Woman and Magic)	2000	France	Not CP
Lady Chatterley	2006	France	Not CP
Late Bloomer	2004	Japan	Cerebral palsy
Le Chiavi Di Casa (The Keys to the House)	2004	Italy	Cerebral palsy
Live Flesh	1997	Spain	Not CP
Livet Är En Schlager (Once In a Lifetime)	2000	Sweden	Cerebral palsy
Losing Will	2007	Canada	Not CP
Magnifico	2003	Philippines	Cerebral palsy
Music Within	2007	USA	Cerebral palsy
My Left Foot	1989	UK	Cerebral palsy
Nationale 7	2000	France	Not CP
Oasis	2002	Korea	Cerebral palsy
Open Hearts	2002	Denmark	Not CP
Orphans	1997	UK	Cerebral palsy
Piedras (Stones)	2002	Spain	Cerebral palsy
Quid Pro Quo	2008	USA	Not CP
ReGenesis/Episode: Race Fever	2008	Canada	Cerebral palsy
Rise and Walk: The Dennis Byrd Story	1994	USA	Not CP
Saved!	2004	USA	Not CP
Say Salaam	2007	India	Cerebral palsy
Secret	2007	Taiwan	Cerebral palsy
Skallagrigg	1994	UK	Cerebral palsy
Some Kind of Miracle	1979	USA	Not CP
Storytelling	2001	USA	Cerebral palsy
Tell Me That You Love Me, Junie Moon	1970	USA	Not CP
Tera Mera Saath Rahen	2001	India	Cerebral palsy
The Bone Collector	1999	USA	Not CP
Le Scaphandre et le Papillon (The Diving Bell and The Butterfly)	2007	France	Not CP
The Kid & I	2005	USA	Cerebral palsy
The People versus Larry Flynt	1996	USA	Not CP
The Score	2001	USA	Cerebral palsy
The Usual Suspects	1995	USA	Cerebral palsy
The Wings of Eagles	1957	USA	Not CP
Touched by Love	1980	Canada	Cerebral palsy
What's Eating Gilbert Grape	1993	USA	Cerebral palsy

**Table 2:** The number of movies in five-year intervals.

	Before 1980	1980–1984	1985–1989	1990–1994	1995–1999	2000–2004	2005–2009	After 2010
Number of movies	1	2	1	3	6	13	7	1

**Table 3:** The incidence of motor types in CP (based on the studies by Howard et al. [6] and Gorter et al. [7]).

Motor types	Incidence in the real world
Spastic	78–86%
Dyskinetic	1.5–6%
Mixed	6.5–9%
Ataxic	3%
Hypotonic	3-4%

**Table 4:** The incidence of motor types of CP in the movies.

Motor types	Incidence in movies (number)	Incidence in movies (percentage)
Spastic	22	65%
Dyskinetic	3	9%
Mixed	9	26%
Ataxic	0	0%
Hypotonic	0	0%

**Table 5:** The incidence of GMFCS levels in CP (based on the studies by Howard et al. [6] and Gorter et al. [7]).

GMFCS levels	Incidence in the real world
GMFCS I	28–35%
GMFCS II	12–16%
GMFCS III	14–19%
GMFCS IV	16–21%
GMFCS V	18–20%

**Table 6:** The incidence of GMFCS levels in CP in the movies.

GMFCS levels	Incidence in movies (number)	Incidence in movies (percentage)
I	14	41%
II	2	6%
III	4	12%
IV	11	32%
V	3	9%

**Table 7:** The comparison of incidence of motor types in CP in the real world and movies.

Motor type	Incidence in the real world	Incidence in movies
Spastic	78–86%	65%
Dyskinetic	1.5–6%	9%
Mixed	6.5–9%	26%
Ataxic	3%	0%
Hypotonic	3-4%	0%

**Table 8:** Comparison of the incidence of GMFCS levels in CP in the real world and in movies.

GMFCS levels	Incidence in the real world	Incidence in movies
I	28–35%	41%
II	12–16%	6%
III	14–19%	12%
IV	16–21%	32%
V	18–20%	9%

**Table 9:** Comparison of the incidence of combined GMFCS levels in CP in the real world and in movies.

GMFCS levels	Incidence in the real world	Incidence in movies
I + II	40–51%	47%
III	14–19%	12%
IV + V	34–41%	41%

**Table 10:** List of movies with GMFCS levels of characters.

Movie title	Year of production	Country of production	Director	Actor	GMFCS level
What's Eating Gilbert Grape	1993	USA	Lasse Hallstrom	Leonardo DiCaprio as Arnie Grape	I

Door to Door	2009	Japan	Takeshi Yoshida	Ninomiya Kazunari as Hideo Kurasawa	I

Secret	2007	Taiwan	Jay Chou	Kuo-zhang Du as Da-jun	II

The Keys to the House (Le Chiavi Di Casa)	2004	Italy	Gianni Amelio	Andrea Rossi as Paolo	III

Inside I'm Dancing	2004	Ireland	Damien O'Donnell	Steven Robertson as Michael Connolly	IV

Oasis	2002	South Korea	Chang-dong Lee	So-ri Moon as Gong-ju Han	V

Skallagrigg	1994	UK	Richard Spence	Adam Walker as Arthur	V

## References

[1] Ivory P. (1997). Disabilities in the media: the movies. *Quest*.

[2] Jarecka U. Desired or real—media images of people with mental disabilities.

[3] http://www.imdb.com/.

[4] http://www.filmweb.pl/.

[5] Marcos M. L. M. (2005). Cerebral palsy in cinema. *Journal of Medicine and Movies*.

[6] Howard J., Soo B., Graham H. K. (2005). Cerebral palsy in Victoria: motor types, topography and gross motor function. *Journal of Paediatrics and Child Health*.

[7] Gorter J. W., Rosenbaum P. L., Hanna S. E. (2004). Limb distribution, motor impairment, and functional classification of cerebral palsy. *Developmental Medicine and Child Neurology*.

[8] Rosenbaum P. L., Palisano R. J., Bartlett D. J., Galuppi B. E., Russell D. J. (2008). Development of the gross motor function classification system for cerebral palsy. *Developmental Medicine and Child Neurology*.

[9] Westbrook M. T., Legge V., Pennay M. (1993). Attitudes towards disabilities in a multicultural society. *Social Science & Medicine*.

[10] Laws G., Kelly E. (2005). The attitudes and friendship intentions of children in United Kingdom mainstream schools towards peers with physical or intellectual disabilities. *International Journal of Disability, Development and Education*.

[11] Colver A. F., Dickinson H. O., Parkinson K. (2010). Access of children with cerebral palsy to the physical, social and attitudinal environment they need: a cross-sectional European study. *Disability and Rehabilitation*.

[12] Scholtes V. A., Becher J. G., Beelen A., Lankhorst G. J. (2006). Clinical assessment of spasticity in children with cerebral palsy: a critical review of available instruments. *Developmental Medicine and Child Neurology*.

[13] Mahant N., McCusker E. A., Byth K., Graham S. (2003). Huntington's disease: clinical correlates of disability and progression. *Neurology*.

[14] Walker F. O. (2007). Huntington's disease. *The Lancet*.

[15] Delgado M. R., Albright A. L. (2003). Movement disorders in children: definitions, classifications, and grading systems. *Journal of Child Neurology*.

